# Green-to-Red Photopolymerization via Dual-Role Dyes for Transparency-Tunable 3D-Printed Hydrogels

**DOI:** 10.1021/acsapm.6c02256

**Published:** 2026-07-31

**Authors:** Ali Eftekhari, Anni Mattila, Shahla Radmehr, Ville Santala, Nikita Durandin, Minna Kellomäki, Timo Laaksonen, Adel Badria

**Affiliations:** † Faculty of Engineering and Natural Sciences, 7840 Tampere University, Tampere 33720, Finland; ‡ Bio- and Circular Economy Unit, Faculty of Engineering and Natural Sciences, Tampere University, Korkeakoulunkatu 8, Tampere 33720, Finland; § Biomaterials and Tissue Engineering Group, Faculty of Medicine and Health Technology, Tampere University, Korkeakoulunkatu 8, Tampere 33720, Finland; ∥ Drug Research Program, Division of Pharmaceutical Biosciences, Faculty of Pharmacy, University of Helsinki, Helsinki 00790, Finland

**Keywords:** red-light photopolymerization, green-light polymerization, hydrogels 3D printing, DLP printing, traceless photopolymerization

## Abstract

Light-based 3D printing typically achieves high speed and resolution through multicomponent photopolymer resins that combine a separate photoinitiator and photoabsorber. Here, we show that a single visible-light dye can perform both roles at once—acting as a photoinitiator and as an optical attenuator—so that print speed, cure depth, resolution, and the final optical clarity of the construct are all governed by one component. The photobleaching and in situ photorheology of five green-to-red dyes (methylene blue, Azure A, thionine, Eosin Y, and Erythrosin B) are compared to establish which dyes combine efficient gelation with near-complete loss of their colored band. Using methylene blue as a model, dye concentration alone is shown to tune critical exposure energy (*E*_c_ = 199–909 mJ cm^–2^), cure depth, transparency, and printed morphology across distinct, reproducible printing regimes, without any auxiliary photoabsorber. Postcured green- and red-light DLP prints become nearly colorless, and their color stability is shown to depend on pH. This single-dye strategy provides a simple, generalizable route to traceless, optically tunable hydrogels for biomedical applications ranging from transparent corneal implants to opaque dermal scaffolds.

## Graphical abstract

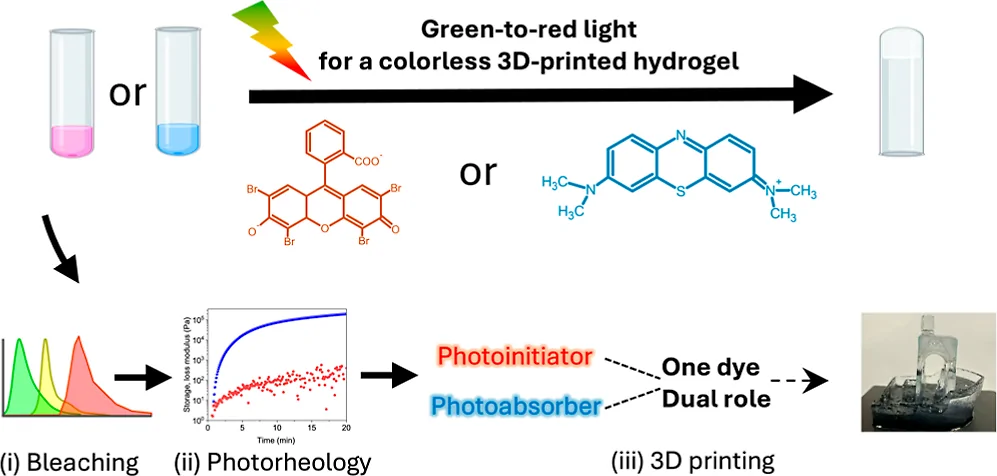

## 1 Introduction

Photopolymerization-based printing enables precise control over reaction dynamics and, at the same time, facilitates the practical implementation of complex structures.^1–3^ Light-triggered polymerization enables on-demand curing of reactive oligomers and is widely used in light-based 3D printing technologies such as stereolithography (SLA) and digital light processing (DLP).^4–8^

Visible-light curing (typically ≥500 nm) more commonly follows a Type II (photoredox) route, in which an excited light-absorbing species generates radicals via electron and/or hydrogen transfer with a co-initiator, while offering improved biocompatibility and deeper penetration than UV irradiation in many polymeric and biological media.^9–16^ Well-known visible light chromophores used as photocatalysts can be grouped into two broad classes: (i) green-light absorbers, such as Eosin Y and Erythrosin B, and (ii) red-light absorbers, such as methylene blue (MB).^17–20^ One of the drawbacks of such visible light chromophores is that they can leave residual color in the cured material; in contrast, traceless (colorless) printing aims to maximize the final optical clarity and improve homogeneous light penetration.^21^ Moreover, in DLP/SLA printing, overcuring must be minimized to preserve both horizontal and vertical resolution and print fidelity.^22,23^ Typically, the photoinitiating system absorbs light at a defined wavelength to generate radicals, while an added photoabsorber (opacifier) limits light penetration depth; the balance between radical generation and optical attenuation governs the print’s resolution, mechanical properties, and optical clarity.^24–27^ In most visible-light Type II systems, the chromophore functions as both the visible-light photoabsorber and the photosensitizer. This dual functionality can reduce formulation complexity by coupling light harvesting and radical generation within the same molecule, thereby decreasing reliance on additional components. However, integrating optical tuning into 3D-printed hydrogels must not compromise printability or resolution: transparent hydrogels (e.g., corneal repair) require high transmission and precise structures, whereas opaque scaffolds (e.g., dermal regeneration) must provide handling contrast while maintaining layer-wise accuracy.^28,29^ In this work, we introduce a visible-light (green-to-red) hydrogel photopolymerization strategy in which the dye plays a dual role as both the light absorber and a sacrificial photoinitiator, eliminating the separate photoabsorber normally required to control cure depth and yielding a colorless hydrogel after curing. This distinguishes the present approach from conventional visible-light DLP formulations, in which light harvesting and optical attenuation are divided between two or more components that must be balanced independently.^30^ It also differs from recent DLP strategies that decouple shape-setting from network completion—for example, combining brief light exposure with subsequent dark polymerization to preserve photoresponsive groups that would otherwise be consumed during printing^31,32^ —and from our earlier demonstration of traceless red-light photopolymerization enabling 3D-printable, cell-laden hydrogels.^21^ In contrast, here we (i) systematically compare five green- and red-absorbing dyes by photobleaching and in situ photorheology and (ii) show that a single parameter—dye concentration—simultaneously sets transparency, cure depth, and resolution. To our knowledge, this is the first systematic single-component framework linking dye photochemistry to the clarity–resolution trade-off across the green-to-red window. Using methylene blue as a model, optical transparency, curing depth, and curing resolution are tuned without auxiliary absorbers, and the effect of environmental pH on postprint color stability is evaluated. Collectively, this study provides a versatile, formulation-simplifying route to traceless hydrogels with application-specific optical and mechanical properties, bridging controllable print fidelity with tailored optical functionality for biomedical applications.

## 2 Materials and Methods

### 2.1 Materials

Poly­(ethylene glycol) diacrylate (PEGDA, average molecular weight 700 Da) was obtained from Sigma-Aldrich (St. Louis, MO, USA). MB (≥97% purity) and triethanolamine (TEA, ≥99%) were purchased from Sigma-Aldrich. Sodium sulfite (Na_2_SO_3_, ≥98%) and hydrochloric acid (HCl, 1 M) were used for oxygen scavenging and pH adjustment experiments, respectively. Ultrapure water (resistivity 18.2 MΩ·cm) was produced using a Milli Q Integral water purification system (Merck Millipore). All chemicals were used as received without further purification.

### 2.2 Preparation of Hydrogel Resins

A 10 mM stock solution of methylene blue was prepared in ultrapure water. Serial dilutions were performed to obtain 15 working concentrations ranging from 5 mM to 0.000304 mM. The standard hydrogel resin consisted of 50 vol % PEGDA, 50 vol % MB working solution, and 1 vol % TEA (relative to total resin volume). pH-adjusted samples were prepared by titrating the resin with 0.1 M HCl to achieve pH 6.3; control samples remained at the native pH of 8.5. All resins were protected from ambient light by using aluminum foil and used within 4 h of preparation.

### 2.3 3D Printing Setup

A custom printing chamber was fabricated to enable single-layer curing studies with minimal resin consumption. A polystyrene cuvette (Brand GmbH) was modified by removing its base and sealing the bottom with a UV transparent glass coverslip (thickness 0.17 mm, Menzel Gläser) using a temporary adhesive (UHU glue stick). A hollow rectangular spacer (internal dimensions: 5 × 5 × 0.3 mm) designed in AutoCAD (Autodesk) and printed using a Formlabs Form 3 printer served as the printing template. The assembly was mounted on the build platform of a Monoprinter MONO3 3CH DLP printer (Monoprinter Oy) equipped with a red and green LED light source (530 and 616 nm). The maximum internal height of the chamber was 30 mm; any cure depth exceeding this limit is reported as >30 mm.

### 2.4 Printing Protocol and Parameter Variation

Printing parameters were controlled via the manufacturer’s slicing software (Monoprinter Studio). Standard printing was performed with a layer thickness of 50 μm, LED intensity of 100% (24.1 mW/cm^2^) unless otherwise stated, and exposure times ranging from 5 to 750 s. For printability optimization, sodium sulfite (0.5 wt %) was added as an oxygen scavenger, and resin was preheated to 50 °C using a dry bath incubator. After each print, uncured resin was removed, and the cured hydrogel layer was carefully detached from the coverslip for analysis.

### 2.5 Characterization and Measurements

Cure depth was measured at three distinct points using a digital depth gauge (Mahr MarCal 16 ER, resolution 0.01 mm, and accuracy ±0.03 mm). The average of three measurements was recorded after subtracting the coverslip thickness. Critical energy curves were generated by plotting cure depth versus curing energy (*E* = intensity × time) and fitting the data to a logarithmic model using Microsoft Excel. Optical documentation was performed using a digital camera (Canon EOS 200D) under consistent lighting. Samples stored under light (ambient laboratory fluorescent lighting) or in darkness were photographed daily for 7 days. pH measurements were taken with a calibrated pH meter (Mettler Toledo SevenCompact). Statistical analysis was performed using GraphPad Prism 9; data are presented as mean ± standard deviation (SD) unless otherwise noted.

### 2.6 Absorption Decay Measurement

Absorbance decay measurements were performed using a Cary 60 UV–Vis spectrophotometer (Agilent Technologies, USA) equipped with a Prior Lumen 1600 illumination source (Prior Scientific, USA) providing narrow-band LED excitation at selected wavelengths. Green irradiation was applied at 525 nm (15 mW cm^–2^) and red irradiation at 635 nm (16.5 mW cm^–2^). Illumination was initiated at *t* = 5 s. Measurements were conducted under both unstirred conditions and with stirring (1000 rpm). The dye concentrations were adjusted to OD = 0.1 for all measurements. The absorption decay traces are representative single measurements (*n* = 1); therefore, no statistical significance testing was performed for these kinetic profiles.

### 2.7 In Situ Photorheology

All rheology measurements were performed using a rotational rheometer (Discovery HR-2, TA Instruments Inc., USA) in a parallel plate geometry with a diameter of 12 mm. For in situ photorheology, Thorlabs LEDs (625 nm, M625L2) and (520 nm, M520L2) equipped with a collimator head was placed at 7 cm from the parallel plate for irradiating the sample during the measurement. Then, for measuring the storage modulus (*G*′) and loss modulus (*G*″), frequency and strain were kept constant at 1 Hz and 0.1%, respectively. To measure viscoelasticity, the frequency was altered from 1 to 100 Hz. For all measurements, the same amount of sample (300 μL) was placed on the parallel plate. The dye concentrations were adjusted to OD = 0.1 for all measurements. The photorheology traces are representative single measurements (*n* = 1); therefore, no statistical significance testing was performed for these profiles.

### 2.8 Fluorescence Emission

The emission signal of dyes was recorded using a Vilber Newton 7 (France) imaging instrument. Azure A and MB were excited at 640 nm, and fluorescence was collected in the 650–700 nm emission window. Thionine was excited at 580 nm and its emission collected in the 620–680 nm emission window. Eosin Y and Erythrosin B were excited at 480 nm, and fluorescence was collected in the 540–560 nm emission window. The emission signal was corrected by the background.

### 2.9 Wet-Mass Swelling Analysis

Wet-mass swelling analysis was performed to evaluate the network integrity of MB-containing PEGDA hydrogels after irradiation/decolorization. Hydrogel precursor solutions were prepared from 40% v/v PEGDA, and MB adjusted to an initial optical density of 0.10, and TEA (37.5 mM). Aliquots of 400 μL were irradiated for 15 min under 660 nm at 30 mW/cm^2^, and the initial wet mass of each cured hydrogel was recorded after gently removing surface liquid. The hydrogels were then immersed individually in Milli-Q water at room temperature. At selected time points, samples were removed, gently blotted to remove excess surface water, weighed, and returned to water. The relative wet-mass change was calculated as

1 $$Relative\;mass\;change\;\left(\%\right)=\frac{W_{t}-W_{0}}{W_{0}}\times 100$$

where *W*_0_ is the initial wet mass after curing and *W*_*t*_ is the wet mass at each time point. Data are reported as mean ± SD from *n* = 3 independent hydrogel samples.

## 3 Results and Discussion

Among thiazine dyes, MB is one of the most widely used and FDA-approved with clinical applications.^33,34^ Azure A and thionine are closely related thiazine dyes that absorb in the red region and share the phenothiazinium core with MB, differing mainly in amino substituents (Figure 1A). In the presence of tertiary amines such as TEA, thiazine dyes undergo photoredox cycling (Figure 1B),^35,36^ and an analogous behavior is observed for green-absorbing xanthene dyes such as eosin Y and erythrosin B.^37^

**Figure 1:**
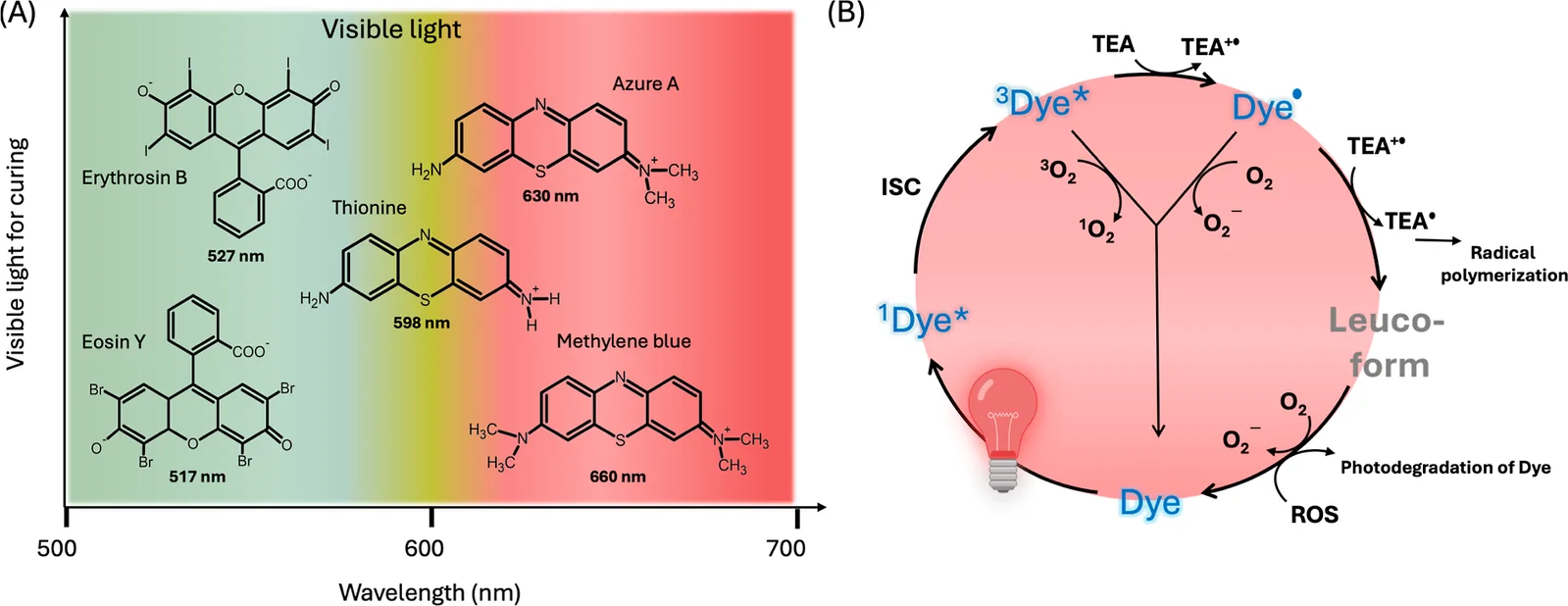
(A) Chemical structures of investigated photoinitiator dyes and their maximum absorption wavelengths in the visible range. (B) Photocatalytic cycle of thiazine dyes upon red-light irradiation in water.

To understand the photobleaching behavior of these dyes, time-dependent decay of dye absorbance was monitored in water under controlled irradiation in ambient conditions. First, the photobleaching of thiazine dyes (MB, Azure A, and Thionine; Figure 2A) in water in the presence of TEA (without PEGDA) was measured under continuous red-light irradiation with an initial dye optical density (OD) of 0.10–0.15, with irradiation initiated at *t* = 5 s (with stirring). As shown in Figure 2A, upon starting irradiation, Thionine exhibited a faster absorbance decay and reached a nonzero plateau sooner than MB and Azure A due to the higher triplet-state oxidation potential (

**Figure 2:**
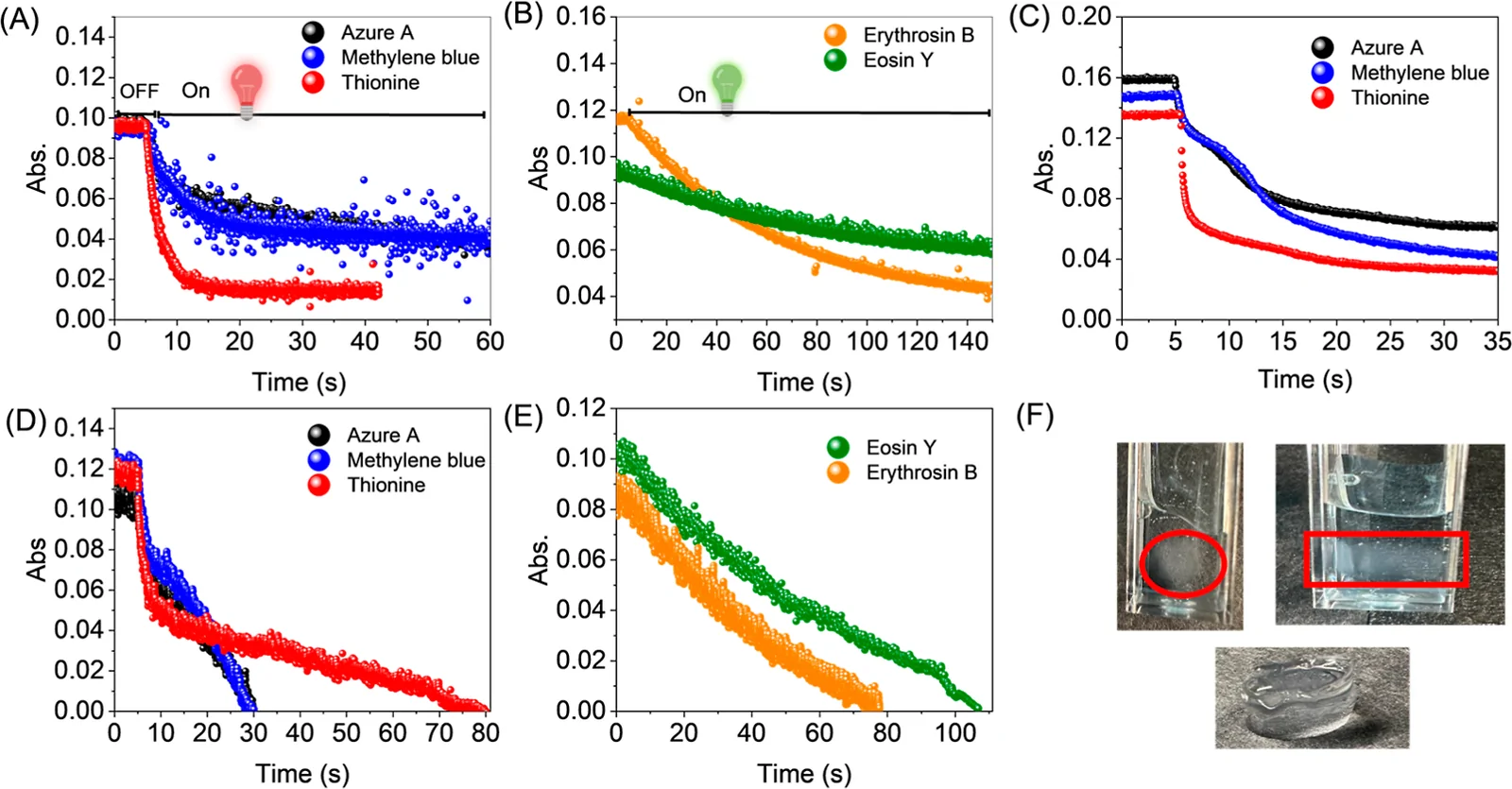
(A) Absorbance decay of MB, Azure A, and Thionine upon red-light irradiation (635 nm) in water with stirring. (B) Absorbance decay of Eosin Y and Erythrosin B upon green-light irradiation (525 nm) in water with stirring. (C) Absorbance decay of Azure A, MB, and Thionine in 40% v/v PEGDA under ambient conditions upon red-light irradiation (635 nm, 15 mW cm^–2^) without stirring. (D) Absorbance decay of Az, MB, and Th in 40% v/v PEGDA (water) under ambient conditions upon red-light irradiation (635 nm, 15 mW cm^–2^) with stirring. (E) Absorbance decay of EY and ErB in 40% v/v PEGDA (water) under ambient conditions upon green-light irradiation (525 nm, 15 mW cm^–2^) with stirring. The stirring speed was maintained at 1000 rpm for all measurements. (F) Showing the localized polymerized region along the light path in the Azure A-containing formulation without stirring. Absorption decay traces are representative single measurements (*n* = 1 per condition).

–1.79 V) as reported by Grossweiner et al.^38^ None of the dyes’ absorbance decayed to zero; instead, a nonzero photostationary absorbance was reached due to TEA-driven reduction and oxygen-driven reoxidation of the dye (Figure 1A).^39,40^

Under green-light irradiation, Eosin Y and Erythrosin B show slower time-dependent absorbance decay than the red-light-absorbing dyes (Figure 2b, in water with stirring). For Eosin Y, this behavior is consistent with oxygen-driven regeneration of the dye dominating over further reduction to the fully reduced leuco form (Figure S1).^41^ The slow absorbance-decay kinetic for Erythrosin B is due to the low efficiency of photobleaching by ^1^O_2_. Also, the intersystem crossing rate constant of Eosin Y was reported as 0.73 × 10^9^ s^–1^, whereas Erythrosin B shows much faster ISC, 6.6 × 10^9^ s^–1^, implying a substantially larger fraction of Erythrosin B singlet state populates its triplet state leading to faster absorbance decay than Eosin Y under the same conditions.^42^

Absorbance decay experiments were measured in the presence of an oligomer (40% v/v PEGDA in water in the presence and absence of stirring). As shown in Figure 2C, without stirring, the absorbance did not decay to zero but instead reached a photostationary plateau due to localized gelation along the illuminated path, which increases viscosity and hinders diffusion (Figure 2F).^43^ In contrast, with stirring the localized gel barrier was eliminated and the absorbance of all dyes decayed toward zero (Figure 2D,E), consistent with improved diffusion. Also, in the presence of PEGDA, the radicals generated by the dye/TEA system are rapidly captured by acrylate polymerization, introducing an irreversible sink that suppresses the photoredox cycling.^16,44,45^

Since absorbance decay does not necessarily track the fate of emissive species (and can be distorted by gel formation), fluorescence emission was monitored to complement absorption measurements and assess residual dye-related signal (Figures S2 and S3). Under red irradiation (37 mW·cm^–2^), MB and Azure A showed rapid signal loss compared to Thionine, decreasing to 3.80% and 4.42% after 5 min and to 0.87% and 0.89% after 15 min, respectively. Under green irradiation (30 mW·cm^–2^), Erythrosin B exhibited a lower fluorescence signal than Eosin Y at all time points; however, its normalized fluorescence decreased less over 15 min (∼21% remaining for Erythrosin B vs–10% for Eosin Y). This behavior is consistent with the higher intersystem crossing efficiency of Erythrosin B, which reduces the emissive singlet-state contribution. These fluorescence measurements indicate that even without stirring, most dyes undergo substantial loss of emissive chromophore under continuous irradiation in 40% v/v PEGDA solution.

To elucidate the reaction kinetics and mechanical evolution during ambient photopolymerization, in situ photorheology was employed. For the red-absorbing dyes, photorheology showed that MB and Azure A initiated polymerization at similar times (≈40 s; Figure 3A), with gelation defined by the crossover *G*′ > *G*″ (Figure S4); however, despite similar photobleaching profiles in 40% PEGDA, Azure A produced an approximately 2-fold higher final *G*′, which may reflect differences in singlet-oxygen quantum yield (MB 0.52 vs Azure A 0.45) and/or aqueous dye aggregation that reduces the photoreactive monomer fraction and effective radical generation.^46–48^ In contrast, Thionine exhibited a longer gelation time than Azure A and MB, likely due to partial inactivation of Thionine-derived initiating species in the PEGDA 40 v/v %, consistent with its absorbance-decay behavior (Figure 2D).^49^ Moreover, although Eosin Y exhibits a slower absorbance decay, it displays faster polymerization kinetics than the other dyes (Figure 3A). This is consistent with regeneration of the ground-state dye during irradiation, enabling Eosin Y to undergo repeated photoactivation cycles and sustain radical generation.^50^ In addition, various concentrations of co-initiator (TEA) on reaction kinetics were measured (Figure 3B–C). Increasing TEA concentration initially accelerated gelation and increased final *G*′. At higher TEA concentration, Azure A showed a nonmonotonic dependence, which may reflect an enhanced bimolecular termination (Figure 3B).^51^ For Eosin Y, increasing TEA concentration to 74 mM accelerated the kinetics, whereas further increases produced little additional change, consistent with saturation of productive quenching and the onset of nonproductive pathways (e.g., cage recombination/back electron transfer and enhanced chain-transfer/radical quenching) that limit the effective initiating-radical yield (Figure 3C).^52,53^ Moreover, Thionine showed a traceless polymerization with both green- and red-light irradiation (Figure 3D,E). Its broad green–red absorption allows excitation at either wavelength, yielding comparable gelation kinetics and a visibly colorless, polymerized light path in both cases (Figure 3E). Together, the photobleaching and photorheology results establish which dyes combine traceless decolorization with sufficient gelation kinetics, motivating the next section where this photochemical balance is translated into printing performance and the clarity–resolution trade-off. Decolorization should not be interpreted solely as destructive dye degradation, since the colored dye can also be photoreduced to a colorless leuco form during the dye/TEA photoredox cycle (Figure 1B). Importantly, our previous work on the MB/TEA/PEGDA system showed by in situ ^1^H NMR that PEGDA acrylate conversion reached ∼60% after 15 min irradiation, confirming that dye-band loss occurs alongside substantial network-forming polymerization rather than at the expense of curing.^54^ Consistent with this, MB hydrogels prepared in the present study showed only moderate wet-mass swelling, reaching 16.17 ± 0.08% at 7 h and 8.96 ± 1.14% at 72 h, with no visible disintegration (Figure S5). These results support that decolorization does not substantially compromise hydrogel network integrity under the tested conditions.

**Figure 3:**
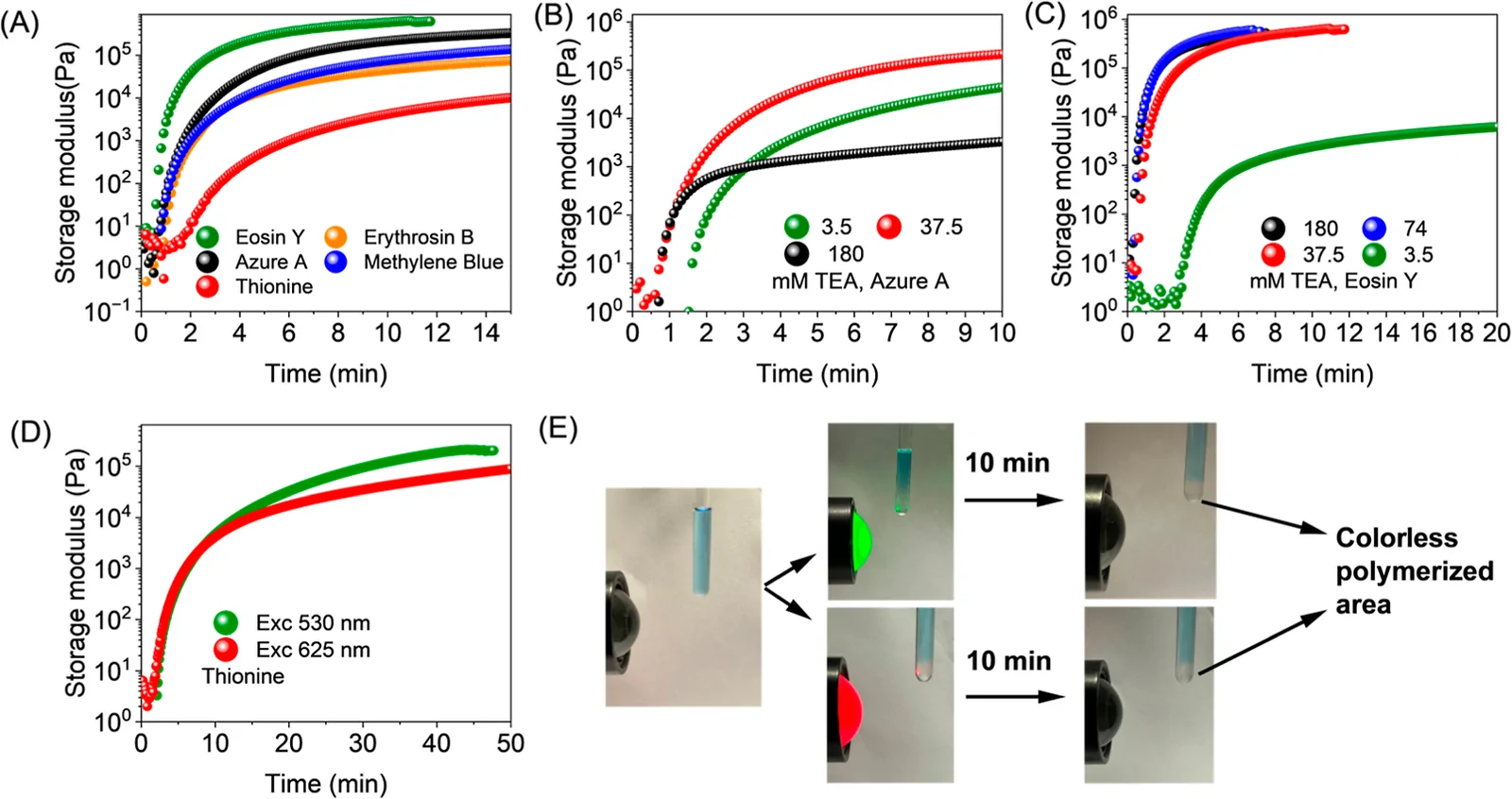
(A) In situ photorheology of green- and red-light photoinitiators upon green- and red-light irradiation. (B) In situ photorheology of Azure A at varying co-initiator (TEA) concentrations under red-light irradiation. (C) In situ photorheology of Eosin Y at varying co-initiator (TEA) concentrations upon green-light irradiation. (D) In situ photorheology of Thionine upon green- and red-light irradiation at the same power density. (E) Thionine photopolymerization under green and red irradiation: in both cases, the illuminated light path polymerized and became colorless. For all experiments, the dyes concentrations were adjusted to OD = 0.1 and the irradiance was set to 30 mW/cm^2^ for both red- and green-light. Photorheology traces are representative single measurements (*n* = 1 per condition).

Light-based 3D printing of colorless biomedical hydrogels (e.g., cornea) using such dyes is often limited by a key trade-off: achieving optical clarity without compromising resolution, mechanical integrity, and/or formulation simplicity. To investigate the aforementioned dual role [photoabsorption (A) vs photoinitiation (I)] of such dyes upon red-light DLP printing, a simple setup was exploited. A stl. design of 0.25 mm^2^ square was repeatedly printed in a custom-made system at different MB concentrations (Figure S6). Such a setup would allow the investigation of the penetration depth, the shape (horizontal resolution), and the opacity of the printed structures using the minimal amount resin and easy-to-handle protocol. To quantify how dye loading alone governs light absorption vs cure depth in our red-light system, the MB concentration was systematically varied. A systematic dilution series of MB (3.0 × 10^–4^ −5.0 mM) revealed distinct optical and curing regimes (Figure 4A). At high concentrations (≥1.250 mM), hydrogels showed an opaque dark blue sharp cube with cure depths below 500 μm. As the MB concentration decreases, transparency increased, with fully transparent and colorless samples achieved at ≤ 0.001 mM MB. Maximum measured cure depth (>30 mm, exceeding the chamber limit, Figure 4A) was observed at 0.039 mM. As the MB concentration decreased, light penetration (and thus cured height) increased up to an optimum. Below this point, penetration continued to increase, but reduced initiation/cross-link density produced mechanically weak gels that collapsed, leading to an apparent decrease in printed height. The relationship between MB concentration and the print height followed a bell-shaped curve, indicating a shift from absorption dominated to initiation limited curing (Figure S7).

**Figure 4:**
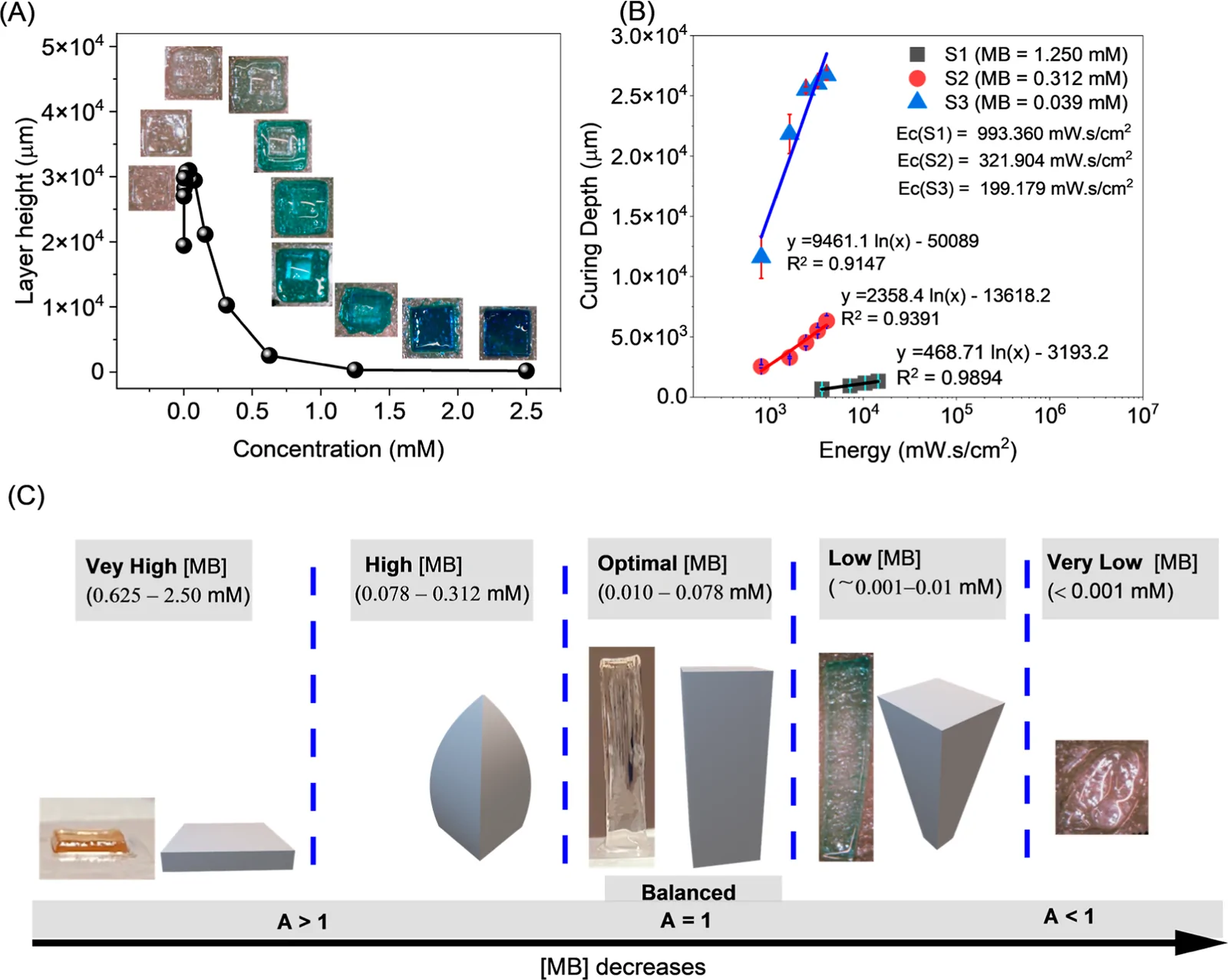
(A) Optical transparency and curing behavior in various concentrations of MB. (B) Critical energy curve at 3 different concentrations of MB (1.25 mM, 0.312 mM, and 0.039 mM). Cure-depth values are shown as mean ± SD from three technical measurements per printed sample (*n* = 3); error bars indicate SD, and solid lines represent logarithmic fits with the corresponding *R* ^2^ values. (C) Printed hydrogel patterns transitioned through four distinct regimes as the MB concentration varied.

The curing behavior of the MB-based formulations was quantified using working curves, where the curing depth is related to the exposure energy to extract the critical energy (*E*_c_) and penetration depth (Dp). The curing behavior *E*_c_ analysis performed at three representative concentrations (1.250, 0.312, and 0.039 mM) demonstrated that the MB concentration directly modulates curing efficiency (Figure 4B). Lower MB concentrations required less energy to initiate polymerization (*E*_c_ = 199 mJ cm^–2^ at 0.039 mM), while higher concentrations increased the energy demand (*E*_c_ = 909 mJ cm^–2^ at 1.250 mM) due to dominant light attenuation. All curing profiles exhibited strong logarithmic fits (*R* ^2^ = 0.9147–0.9903), confirming classic photopolymerization kinetics (Figure 4B). The slope of the cure depth energy curve decreased with increasing MB concentration, visually capturing the transition from penetration sensitive to absorption limited curing. This result clearly confirms that the MB concentration directly dictates both the energy required for gelation and the depth of light penetration, validating its dual function behavior. The relationship between MB concentration and *E*_c_—where higher concentrations increase *E*_c_ due to dominant absorption—provides a predictive framework for resin optimization. This eliminates the need for iterative dye initiator balancing, streamlining formulation for applications ranging from transparent corneal implants to opaque dermal scaffolds.

Upon varying MB concentration vs minimum printing time of single square print, produced five reproducible printing morphologies, reflecting systematic shifts in photoabsorption vs radical generation (Figure 4C). At very high MB loadings (2.5–0.625 mM), printed features adopted a sharp square with very limited height <700 μm, indicating strong light attenuation effect vs radical generation. At concentration (0.312–0.078 mM), a tapered, pillar-like geometry with wide square base, and pointed tip, consistent with increasing radical formation (and polymerization) still, the photoabsorption effect of the MB is still dominant. At an intermediate (“optimal”) MB concentration (0.078–0.010 mM), curing became more uniform across the voxel, yielding high-fidelity cuboids with sharp edges and minimal distortion indicating equal driving forces for resolution control. When MB was reduced to low concentrations (0.001–0.01 mM), features transitioned to an inverted-taper morphology (narrow square base, wider square top), consistent with lower light attenuation to the projected diffusing square-shaped light (lower horizontal resolution) and increase in the radical generation in the projection direction. Finally, at very low MB (≤0.0006 mM), only collapsed or amorphous deposits formed, indicating insufficient cross-link density to maintain mechanical integrity during printing and postprocessing. Collectively, these regimes provide a visual map of MB’s shifting function in the resin: at high concentrations, MB primarily acts as a light attenuator, enforcing shallow curing and tapered profiles, whereas at low concentrations, the system becomes increasingly initiation-limited, leading to overpenetration, scattering-induced broadening, and ultimately mechanical failure. Importantly, these transitions are direct manifestations of light–matter interactions rather than printing artifacts and therefore offer a practical design rule: MB concentration can be selected to balance transparency and cure depth with geometric fidelity, depending on the targeted scaffold architecture.

The effect of exposure time on such formed regimes showed that increasing the light exposure for different concentrations, e.g. 2.5 mM, from 150 s (the minimum required) to 600 s of 660 nm light did not show a difference in the final regime only on the height (from 700 to 1250 μm) of the cured shape. The same was observed for 0.078 mM 12 s (minimum required) to 600 s.

Oxygen is a well-known inhibitor of acrylate radical photopolymerization because it rapidly converts carbon-centered radicals into relatively unreactive peroxyl radicals, delaying gelation until dissolved oxygen is depleted. Accordingly, introducing an oxygen scavenger (e.g., sulfite) and elevating temperature—both of which reduce the impact of oxygen and accelerate curing/transport—are established strategies to shorten inhibition and improve vat-photopolymerization performance. To improve short-exposure printability under ambient conditions (lower than 12 s/layer for 0.039 mM of MB), an oxygen-scavenging and thermal-assistance strategy was evaluated. Adding 0.5 wt % sodium sulfite and preheating the resin to 50 °C reduced the minimum reliable layer time from 12 to 10 s, while maintaining dimensional fidelity. At 5 s exposure, features were still gelled but became irregular, indicating that initiation occurred but network build-up remained insufficient for accurate layer replication. In contrast, increasing TEA concentration over the 0.037 mM produced no meaningful improvement at short layer times, consistent with oxygen inhibition—rather than co-initiator availability—being the dominant limitation under these conditions.

To assess postprint color stability, the pH dependence of MB-containing hydrogels was evaluated with no postcuring (Figure S8). Under identical ambient light and oxygen conditions, MB-containing hydrogels at pH 8.5 faded to a transparent, faintly green appearance within 24 h, whereas samples at pH 6.3 became transparent but brown-tinted. In contrast, samples stored in the dark retained their original blue coloration at both pH values. In addition, time-dependent color evolution showed a strong dependence on the initial MB loading. High-MB concentration (≥0.625 mM) retained a blue appearance for >72 h before transitioning through green to brown, whereas intermediate concentrations (0.078–0.312 mM) faded within 24–48 h and exhibited pronounced yellow intermediates. At low MB (≤0.039 mM), the blue color disappeared within 24 h with minimal yellowing. Overall, the sequence (dark blue → faint/transparent blue → colorless → yellow → brown) is consistent with an oxygen/ROS-driven, multistep photodegradation process of phenothiazinium dyes, in which partially demethylated intermediates (azure/thionine-type species) can transiently contribute to blue–green hues before further oxidation yields yellow/brown products.

Representative DLP prints of a standardized “boat” benchmark (Figure 5) were produced from PEGDA (40% v/v in water) using either red-absorbing thiazine dyes (Azure A, MB, and Thionine; all with TEA = 37 mM) and green-absorbing xanthene dyes (Erythrosin B and Eosin Y; both with TEA = 75 mM). For the red-light formulations, printing was performed at 50 μm layer thickness and 7 mW·cm^–2^ (616 nm) using OD = 3.0 (Azure A: 90 μM, 10 s/layer; MB: 60 μM (in the same range of optimal concentration as discussed in the previous part), 15 s/layer; thionine: 165 μM, 10 s/layer). In all three cases, the global hull geometry and sharp macroscopic edges were reproduced, whereas fine recessed features (chimney and window openings) exhibited localized overcuring, consistent with cumulative dose and light scattering in confined regions. After postcuring (15 min, red light), the prints transitioned from strongly colored to nearly colorless, with thionine retaining only a very faint yellow tint. For the green-light formulations, prints were obtained at 50 μm layer thickness and 37 mW·cm^–2^ at 530 nm (Erythrosin B: 12.5 μM, 3.6 s/layer; Eosin Y: 12.5 μM, 3.5 s/layer). These conditions likewise yielded well-defined boat geometries with sharp edges, while the chimney and windows again showed mild over polymerization; following postcuring (15 min, green light), both xanthene-based prints became largely transparent with a faint yellow residual hue. Increasing dye concentration generally improves print resolution by enhancing optical attenuation and reducing cure-through; however, it also increases residual coloration and typically requires longer postcuring to achieve complete decolorization. To quantify the dimensional fidelity of the DLP prints, the X, Y, and Z dimensions of representative postcured boat structures were measured from calibrated images using ImageJ and compared with the corresponding STL dimensions (Section S9, Table S1). Across the five dye systems, the mean absolute dimensional error ranged from 3.07% to 6.62%, indicating that the global boat geometry was reproduced with moderate fidelity. Most deviations were positive, consistent with mild feature enlargement caused by light scattering/cure-through and localized overcuring, in agreement with the observed overpolymerization in fine recessed features such as the chimney and windows. Beyond dimensional fidelity and optical performance, the suitability of these printed hydrogels for biomedical applications also depends on the cytocompatibility of the complete formulation and curing conditions. The MB/TEA photoinitiating chemistry has previously supported NIH-3T3 fibroblast viability and proliferation in cell-laden hydrogels,^21^ and scaffolds prepared from 50% PEGDA using a visible-light dye/TEA system have also been reported to support endothelial-cell viability.^55^ However, the exact PEGDA/dye/TEA formulations and curing conditions used in the present study were not directly evaluated with cells. Formulation-specific cytocompatibility testing is therefore required before biomedical application.

**Figure 5:**
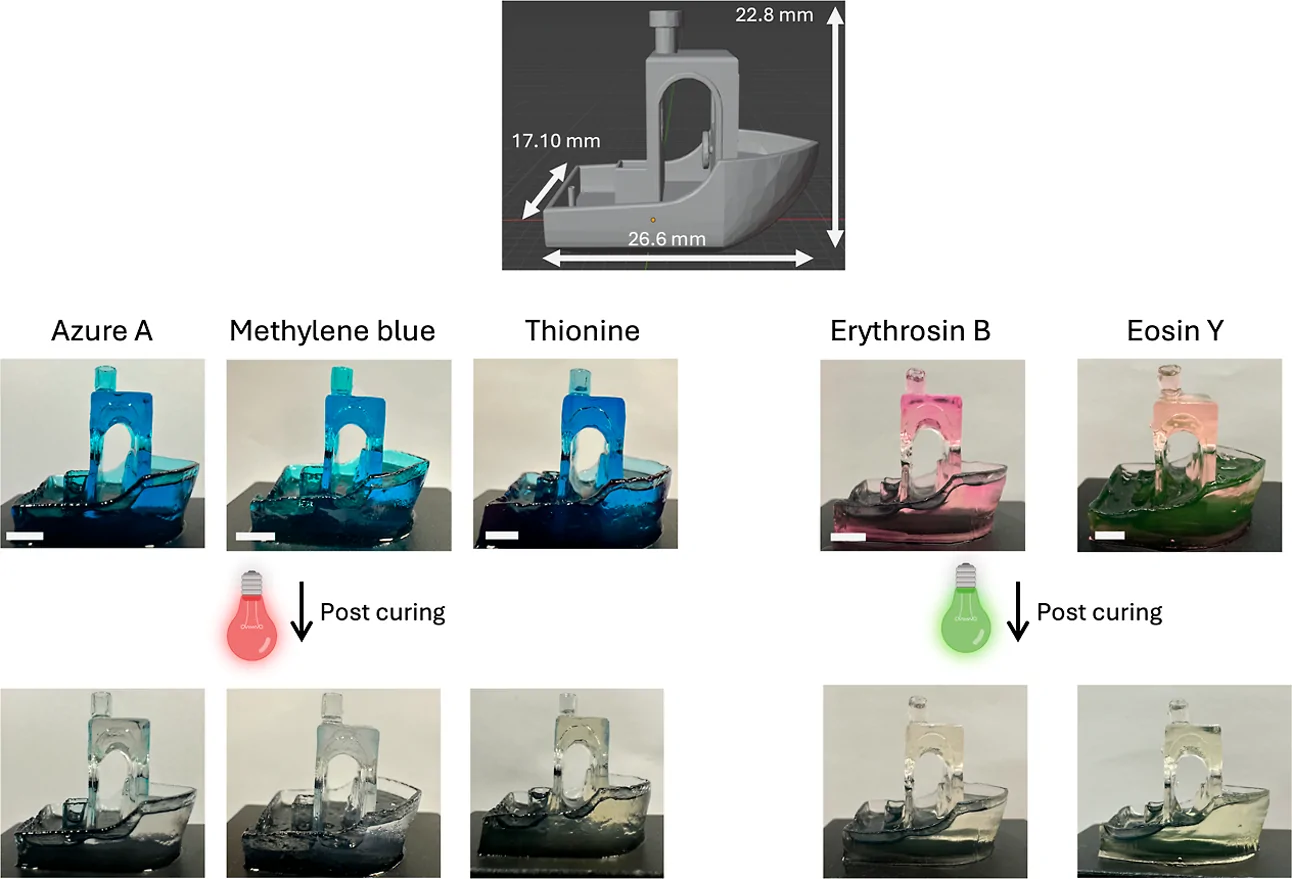
DLP printing of a standardized “boat” benchmark using red- and green-light dye/TEA photoinitiating systems (50 μm layer thickness). Top: CAD model with nominal dimensions. Middle: as-printed boats fabricated with Azure A, MB, and Thionine (red absorbers) and Erythrosin B and Eosin Y (green absorbers). Bottom: corresponding prints after postcuring, showing substantial loss of initial dye coloration. Printing conditions: red-light (616 nm, 7 mW·cm^–2^; PEGDA 40% v/v; TEA 37 mM; OD = 3.0; Azure A 90 μM, 10 s/layer; MB 60 μM, 15 s/layer; thionine 165 μM, 10 s/layer; postcure 15 min red-light 40 mW/cm2) and green-light (530 nm, 27 mW/cm2; TEA 75 mM; OD = 1.0; Erythrosin B 12.5 μM, 3.6 s/layer; Eosin Y 12.5 μM, 3.5 s/layer; postcure 15 min green-light 30 mW/cm2). Scale bar 5 mm.

## 4 Conclusion

In this study, a single visible-light dye was shown to act simultaneously as the photoinitiator and photoabsorber, providing a formulation-simplifying route to traceless hydrogels. Across both green- and red-absorbing dyes, photoredox initiation was coupled to progressive loss of the colored ground-state band during network formation, enabling postcure decolorization under ambient conditions. Photobleaching and in situ photorheology clarified how oxygen transport, diffusion (stirring), and co-initiator (TEA) concentration govern the transition from reversible photostationary cycling to near-complete dye-band loss in oligomer-containing formulations; the systematic dependence of gelation on TEA concentration confirms that radicals are generated through the dye/amine photoredox pathway rather than by passive absorption alone. Using MB as a model, dye concentration alone provided a single, predictive handle over the working-curve metrics—critical exposure energy (*E*_c_ = 199–909 mJ cm^–2^), cure depth, transparency, and printed morphology across reproducible regimes—without added opacifiers. Finally, the pH-dependent color evolution of the printed hydrogels emphasizes that environmental conditions must be considered when targeting long-term optical stability. Together, these results establish dye concentration as a versatile design parameter for balancing cure depth, decolorization, and print fidelity, offering a simple and generalizable strategy for optically tunable, traceless visible-light printed hydrogels for biomedical applications.

## Supplementary Material



## Notes

Photocatalytic cycle of Eosin Y; fluorescence emission decay of thiazine dyes under red light and of xanthene dyes under green light; loss-modulus photorheology traces; swelling behavior of the MB-containing PEGDA hydrogel; custom single-layer DLP printing setup; relation between MB concentration and print height; pH-dependent coloration of hydrogel samples; and dimensional fidelity of DLP-printed boat structures with different dyes (PDF)
