# Supplementary material for: Green-to-Red Photopolymerization via Dual-Role Dyes for Transparency-Tunable 3D-Printed Hydrogels
Source: ACS Appl Polym Mater. 2026 Jul 31;8(15):13267–76. doi: 10.1021/acsapm.6c02256 (PMC13491552; doi:10.1021/acsapm.6c02256)
Supplement: Supplementary file 1 [file ap6c02256_si_001.pdf]

Supporting Information

Green-to-red photopolymerization via Dual-Role Dyes for Transparency-Tunable 3D-Printed Hydrogels

Ali Eftekhari*[1], Anni Mattila[1], Shahla Radmehr [2], Ville Santala [2], Nikita Durandin[1], Minna Kellomäki [3], Timo Laaksonen*[1,4], Adel Badria*[1]

[1] Faculty of Engineering and Natural Sciences, Tampere Univer-sity, 33720 Tampere, Finland. [2]Bio- and Circular Economy Unit, Faculty of Engineering and Natural Sciences, Tampere University, Korkeakoulunkatu 8, 33720 Tampere, Finland. [3] Biomaterials and Tissue Engineering Group, Faculty of Medicine and Health Technology, Tampere University, Korkeakoulunkatu 8, 33720 Tampere, Finland. [4] Drug Research Program, Division of Pharmaceutical Biosciences, Faculty of Pharmacy, University of Helsinki, Helsinki, 00790, Finland.

The corresponding authors email (ali.eftekhari@tuni.fi; timo.laaksonen@helsinki.fi; Adel.Badria@tuni.fi)

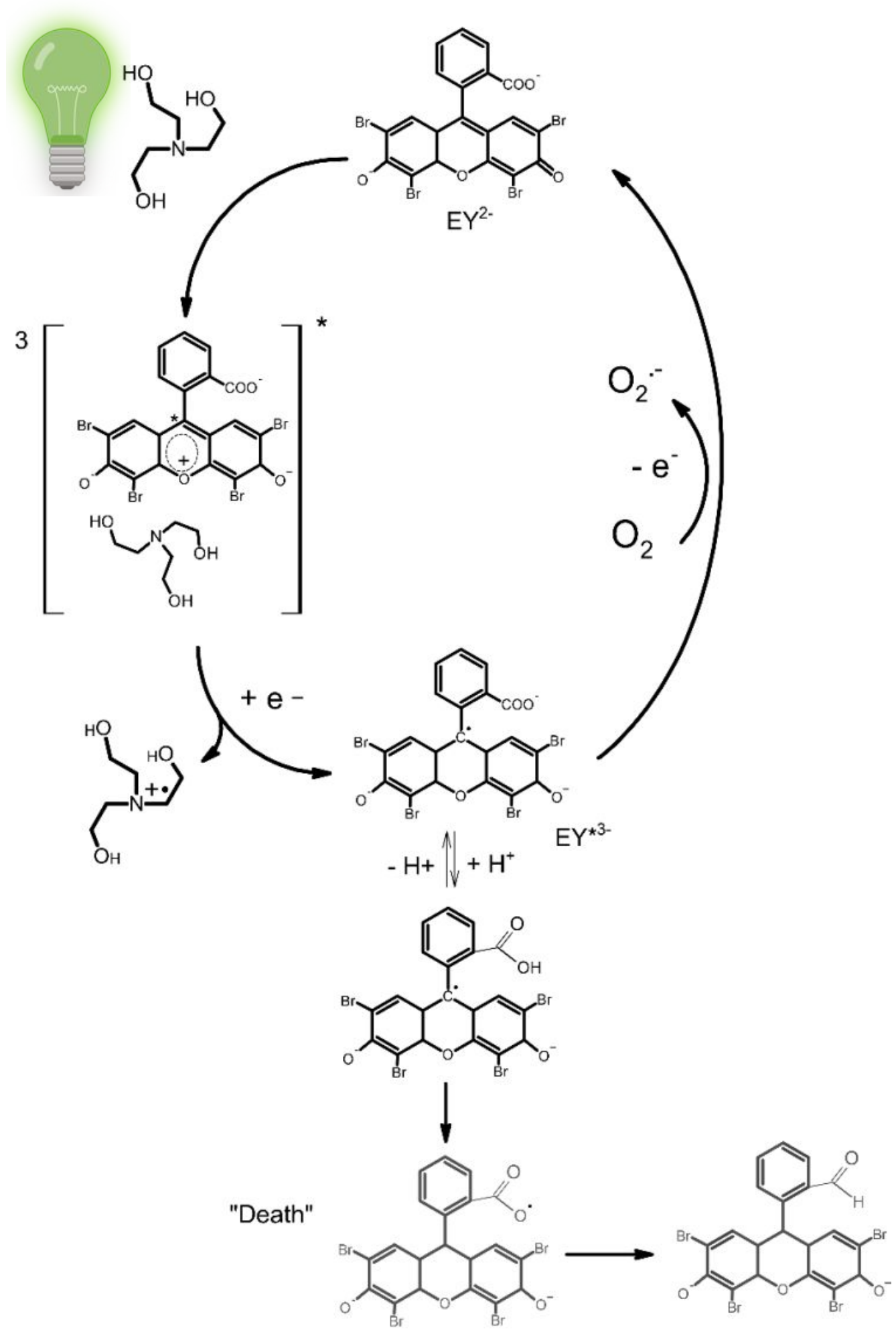

Figure S1. Photocatalytic cycle of Eosin Y upon green-light irradiation. Eosin Y can be reduced to a "temporary" reduced form (EY·3-/EY-H·2-, Figure S1) that oxygen can efficiently convert back to ground (colored) Eosin Y. Therefore, the visible absorbance does not drop much. A lasting absorbance decrease occurs only when the dye is further reduced to the photoinactive leuco form, which is slow to re-oxidize . Because formation of this leuco species competes with oxygen-driven dye regeneration, the overall absorbance decay can be slow.

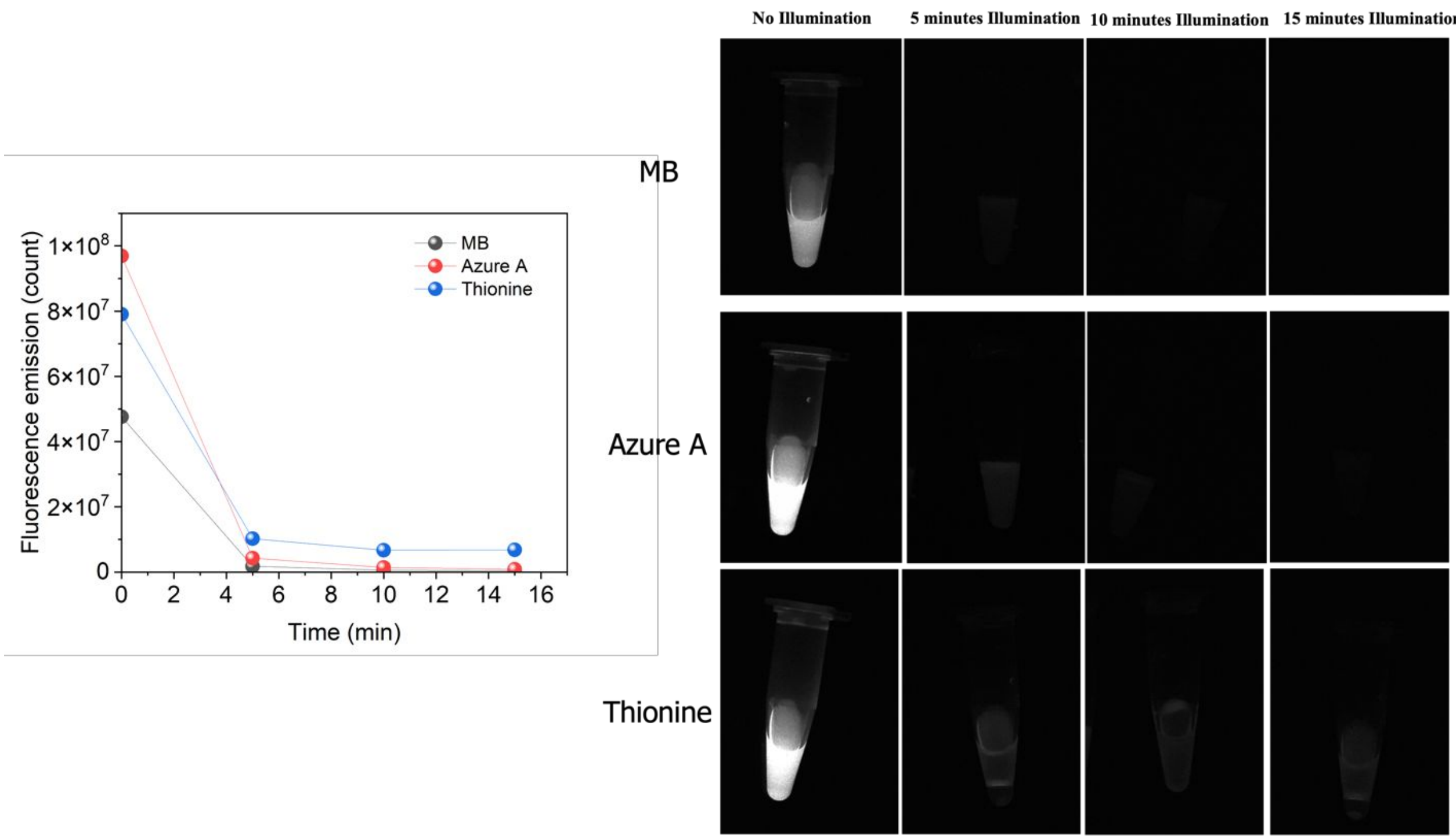

Figure S2. Fluorescence emission of thiazine dyes (Y-axis) during continuous red-light irradiation (Time, X-axis). The fluorescence signal decreased rapidly and approached a plateau after ~5 min, with >95% signal loss for Azure A and methylene blue and ~87% loss for Thionine, indicating substantial conversion of the emissive chromophore. In this experiment, the entire solution volume was uniformly irradiated. These results show that, even without stirring, most of the dye signal is lost under prolonged irradiation. Azure A and MB were excited at 640 nm, and fluorescence was collected in the 650–700 nm emission window. Thionine was excited at 580 nm and its emission collected in 620-680 nm emission window. The emission signal corrected by the background. All dyes OD considered to be 0.1in 40% v/v PEGDA.

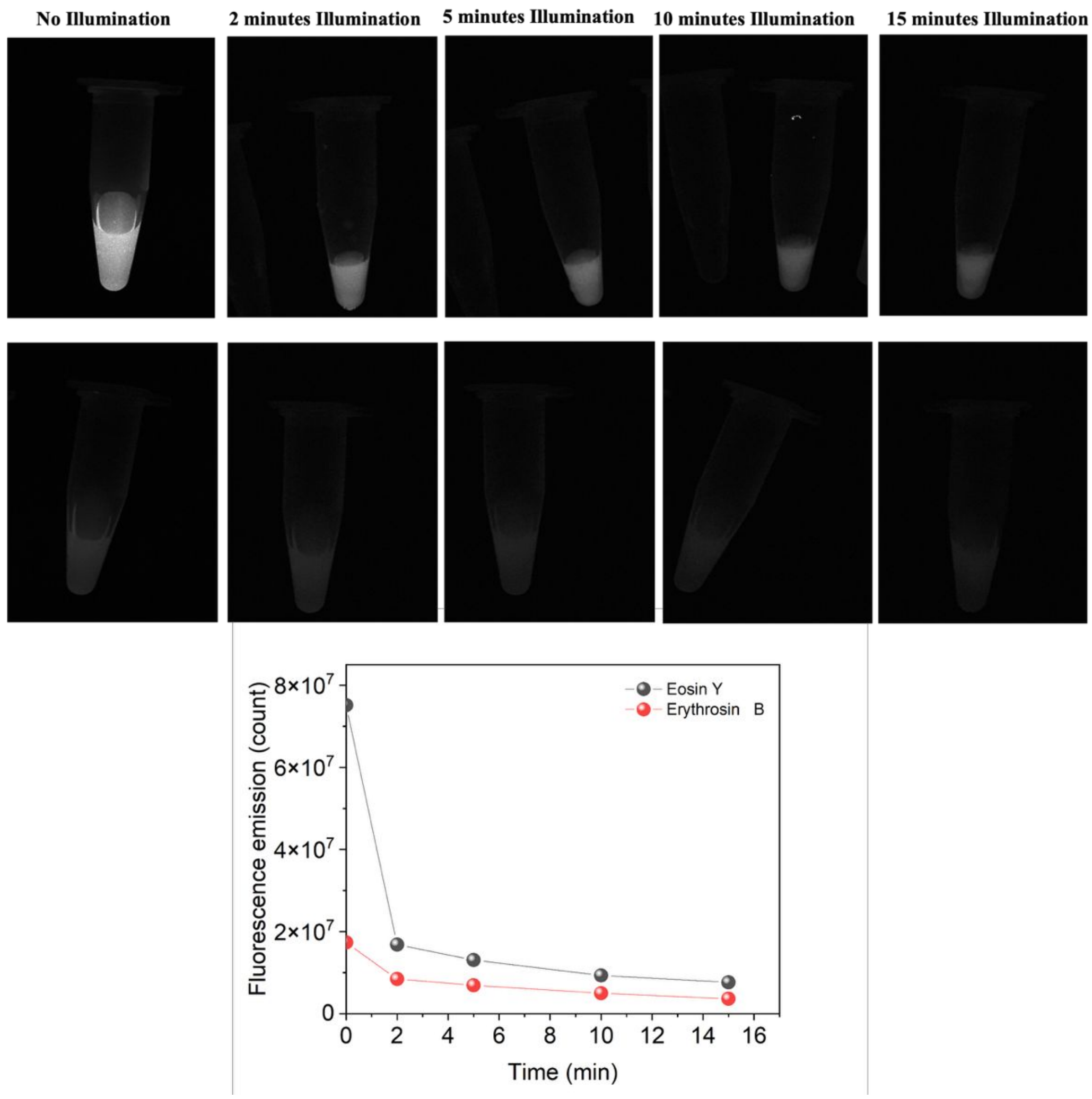

Figure S3. Fluorescence emission of Eosin Y and Erythrosin B (Y-axis))during continuous green-light irradiation (X-axis). The fluorescence signal decreased rapidly and approached a plateau after ~2 min, with >85% signal loss for Eosin Y and ~52% loss for thionine, indicating substantial conversion of the emissive chromophore. In this experiment, the entire solution volume was uniformly irradiated. These results show that, even without stirring, most of the dye signal is irreversibly lost under prolonged irradiation. Eosin Y and Erythrosin B were excited at 480 nm, and fluorescence was collected in the 540-560 nm emission window. The emission signal corrected by the background. All dyes OD considered to be 0.1in 40% v/v PEGDA.

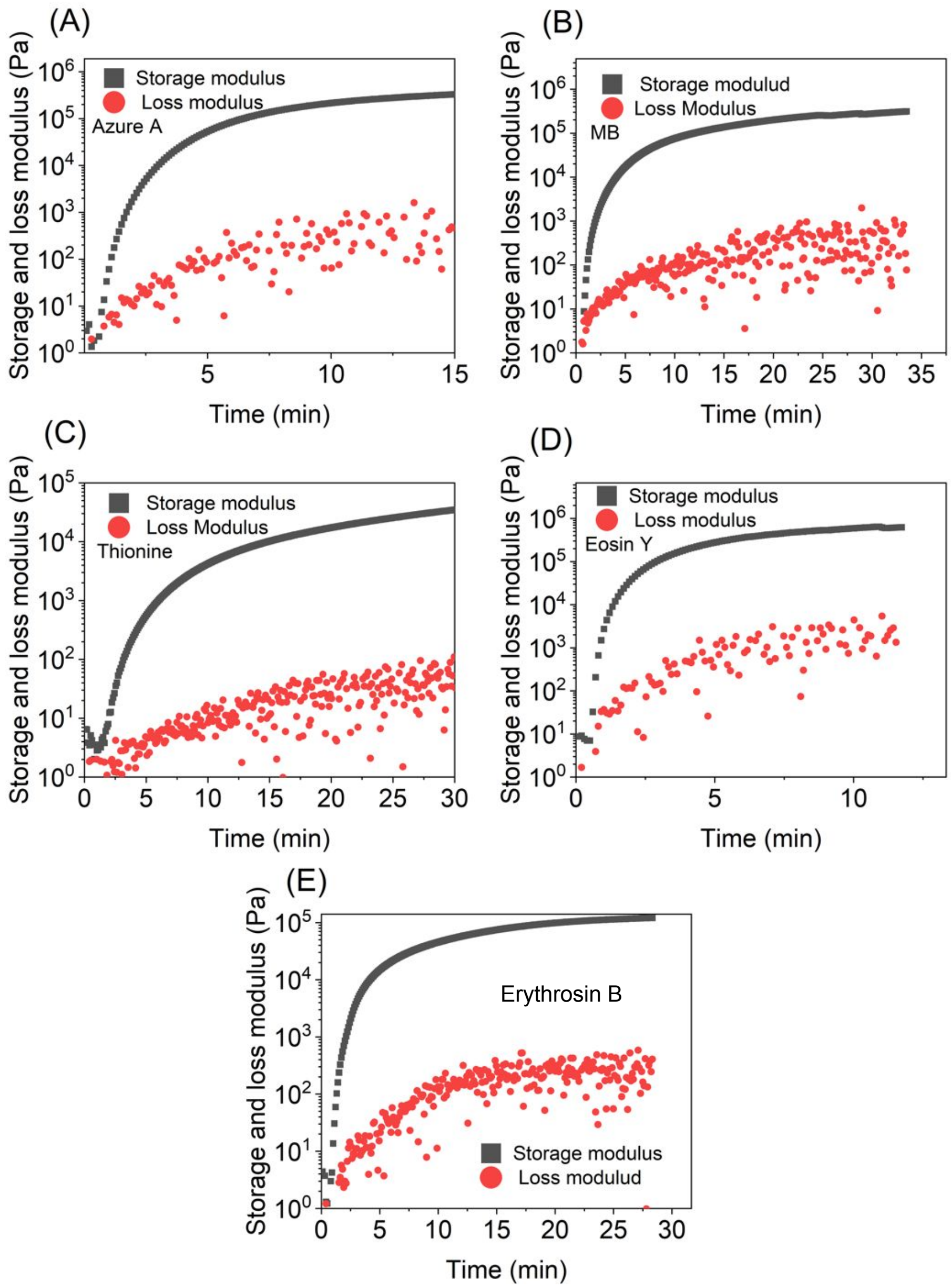

Figure S4. In situ photorheology of green- and red-light photoinitiators upon green- and red-light irradiation with their loss modulus. The gelation time was defined as the point at which the storage modulus (G′) exceeded the loss modulus (G″).

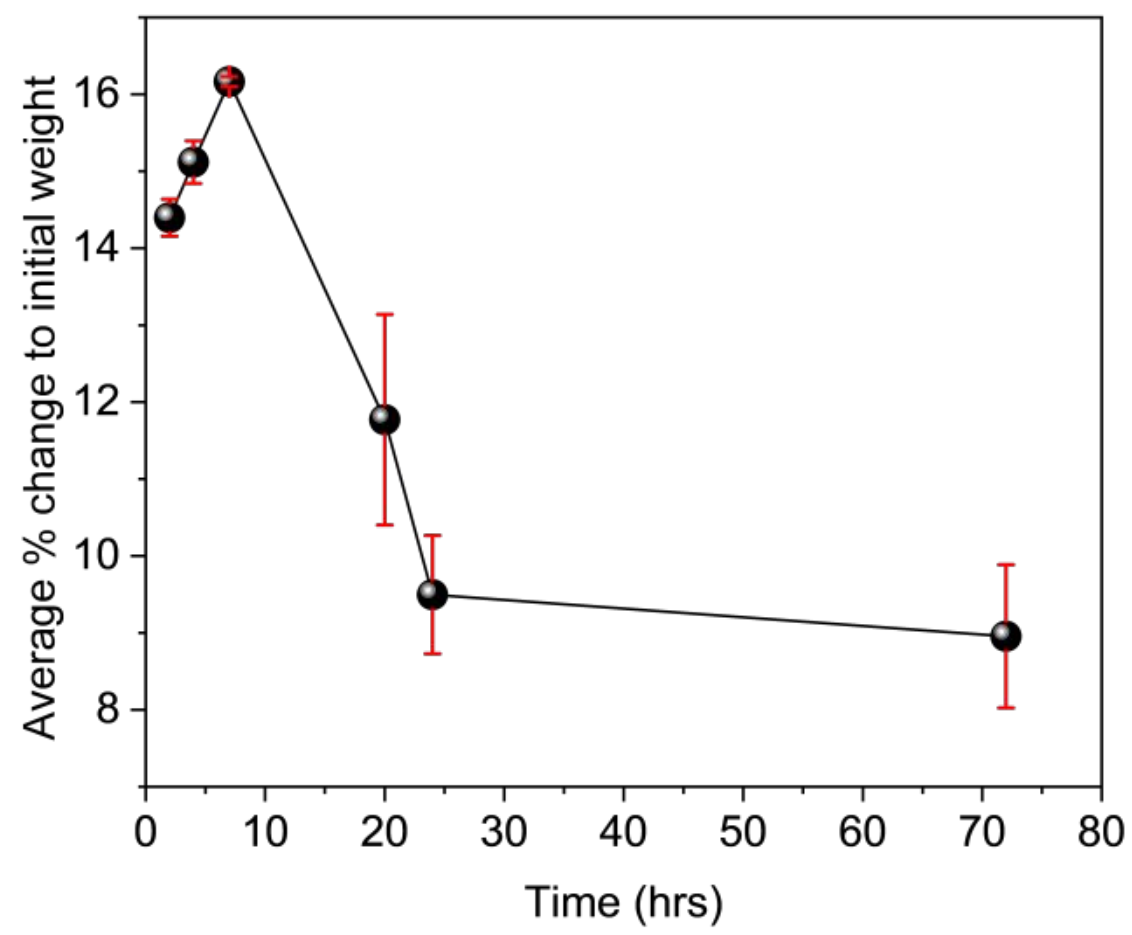

Figure S5. Wet-mass swelling behavior of MB-containing PEGDA hydrogels after irradiation. Relative mass change of cured hydrogels prepared from 40% v/v PEGDA, MB (OD = 0.10), and TEA (37.5 mM) after 15 min irradiation at 660 nm (30 mW/cm^2), followed by immersion in water. The hydrogel mass increased during the first 7 h, reaching a maximum swelling of ~16%, and then partially equilibrated to ~9% after 72 h. Data are shown as mean ± SD, n=3.

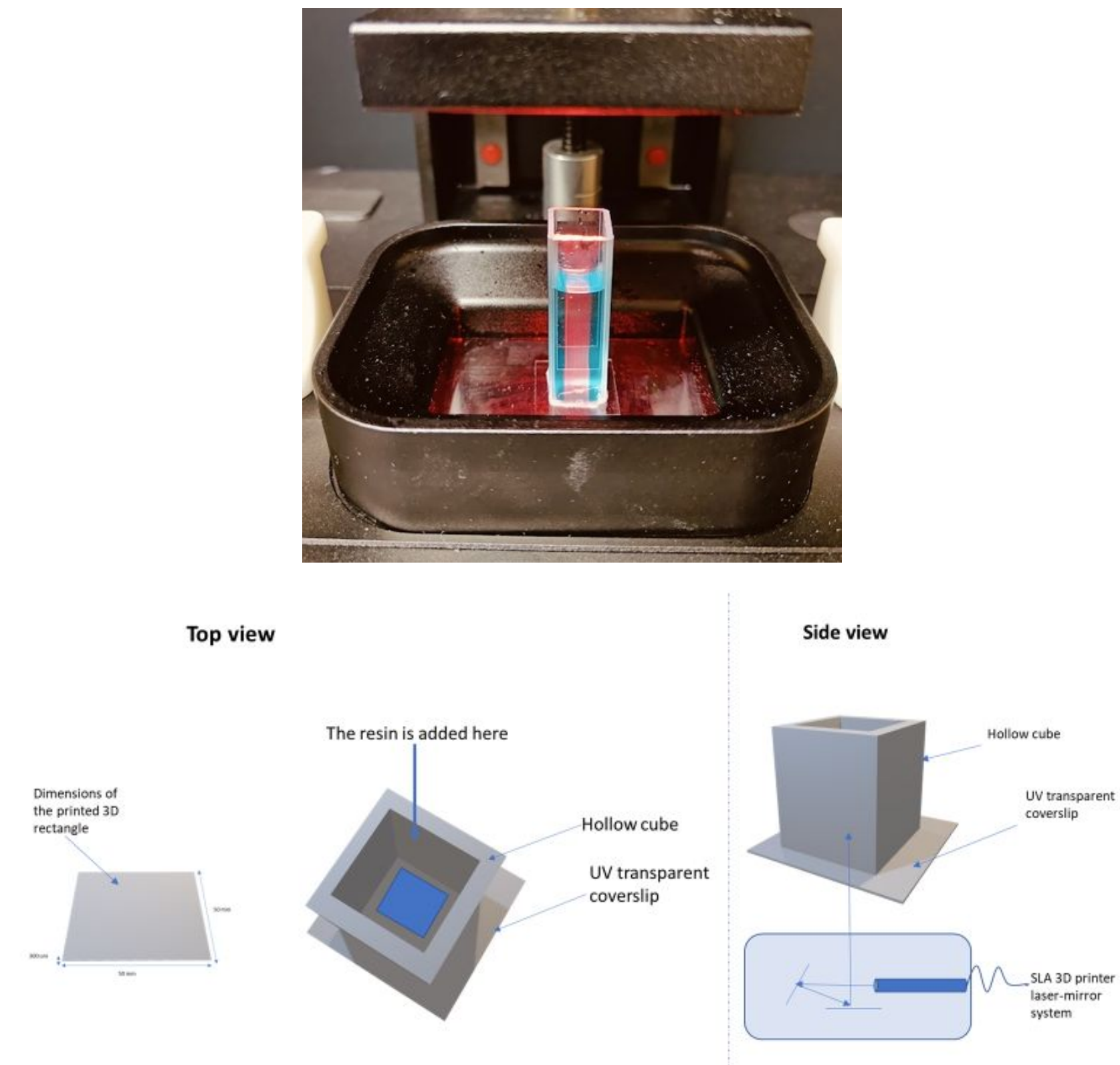

Figure S6. Custom-made setup printing setup for single-layer DLP printing. Using AutoCAD, a simple rectangle was designed with dimensions of 5 x 5 x 0.3 mm (l, w, t). Different formula curing depth and critical energy were tested using the setup shown below. A transparent coverslip with a hollow cube was settled on the top, this setup was filled with the formula resin of interest and one layer was printed and measured. The different measurements (three reach) were done using a digital caliper.

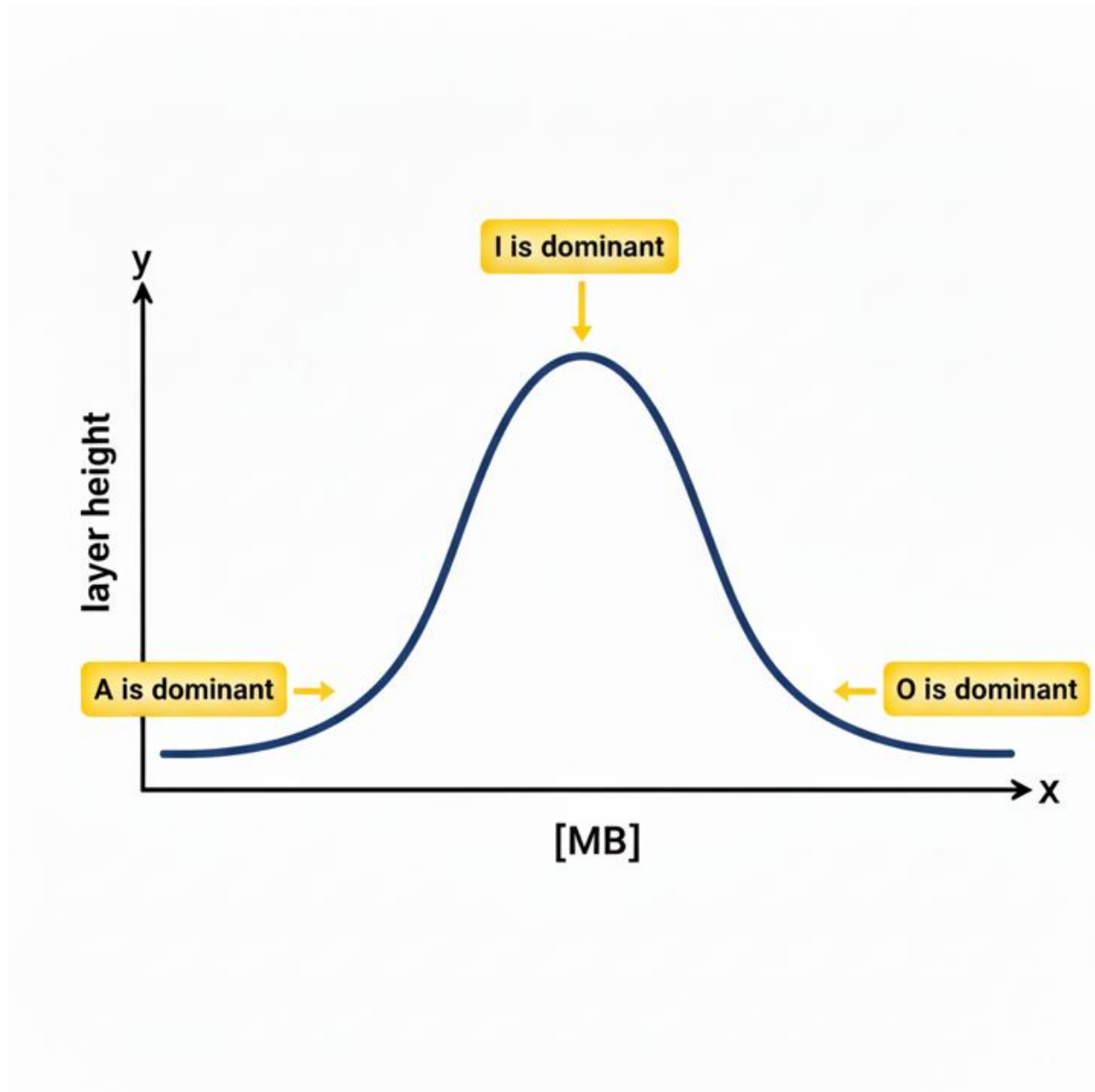

Figure S7. The relation between MB concentration and layer (print) height.

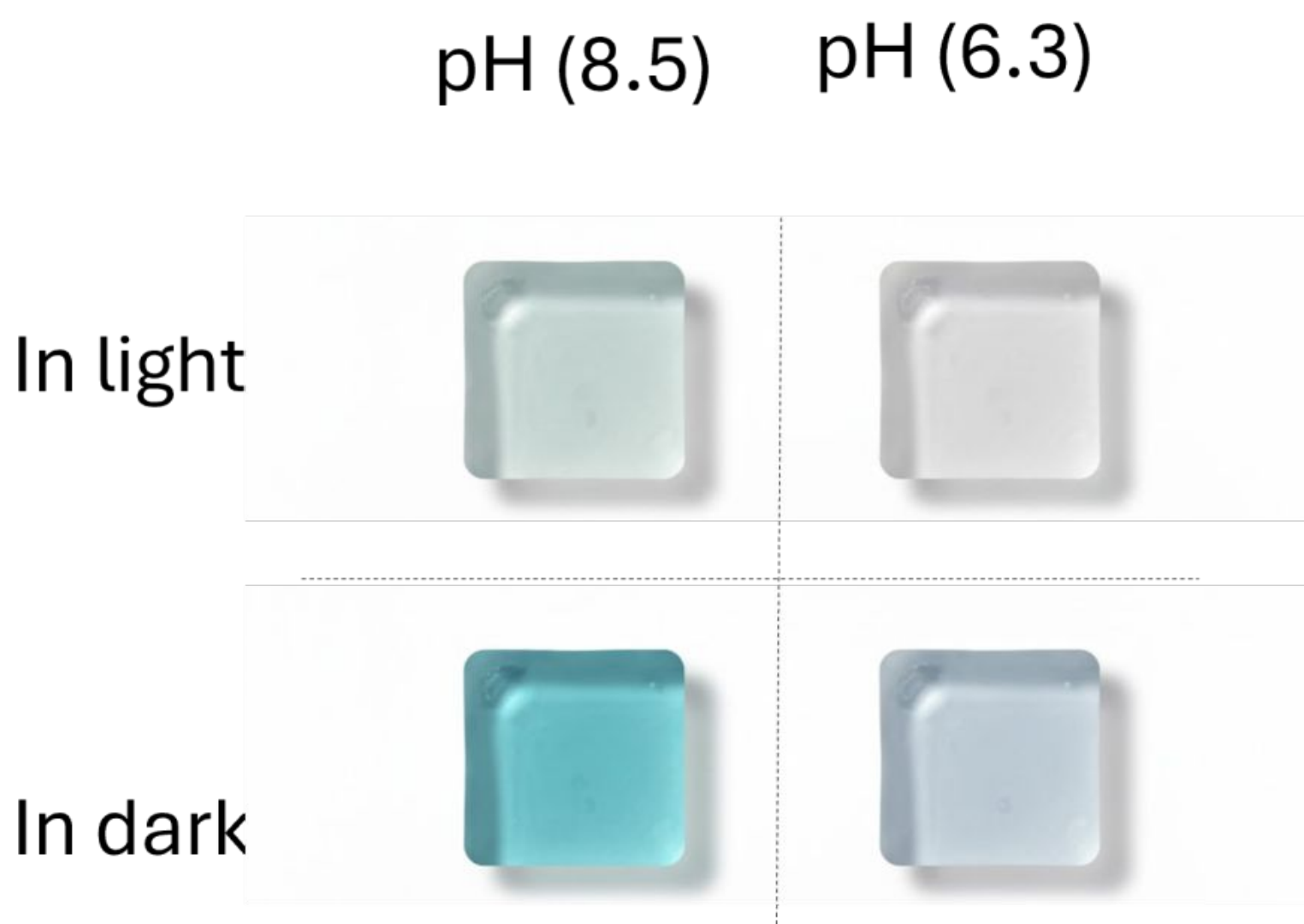

Figure S8. Hydrogel samples with pH-dependent coloration with no post curing. The experiment performed under ambient room light.

Table S1. Dimensional fidelity of DLP-printed boat structures prepared with different dye/TEA photoinitiating systems. The printed X, Y, and Z dimensions were measured from calibrated images using ImageJ and compared with the corresponding STL dimensions (X = 26.60 mm, Y = 17.10 mm, Z = 22.80 mm). Each dimension was measured three times on one representative printed structure per dye, and values are reported as mean ± SD. Dimensional error was calculated as $(D_{\text{printed}} - D_{\text{STL}})/D_{\text{STL}} \times 100\%$. Positive values indicate feature enlargement relative to the STL model.

Dye	X mean ± SD (mm)	X error (%)	Y mean ± SD (mm)	Y error (%)	Z mean ± SD (mm)	Z error (%)	Mean absolute error (%)
**MB**	28.47 ± 0.06	+7.03	18.36 ± 0.15	+7.37	23.77 ± 0.09	+4.27	6.22
**Azure A**	27.06 ± 0.19	+1.72	19.06 ± 0.11	+11.46	24.33 ± 0.40	+6.70	6.62
**Thionine**	28.22 ± 0.29	+6.08	18.44 ± 0.18	+7.84	23.51 ± 0.09	+3.11	5.68
**Erythrosin B**	26.76 ± 0.16	+0.61	17.59 ± 0.22	+2.87	24.10 ± 0.18	+5.72	3.07
**Eosin Y**	27.49 ± 0.27	+3.36	17.35 ± 0.17	+1.46	24.62 ± 0.24	+7.98	4.27
